# Factors Associated with Late Diagnosis of Human Immunodeficiency Virus/Acquired Immunodeficiency Syndrome (HIV/AIDS) in a University Hospital in Brazil: Challenges to Achieving the 2030 Target

**DOI:** 10.3390/v15102097

**Published:** 2023-10-17

**Authors:** Ligia Maria Nascimento Arantes, Andrey Oeiras Pedroso, Mayra Gonçalves Menegueti, Elucir Gir, Eliã Pinheiro Botelho, Ana Cristina de Oliveira e Silva, Renata Karina Reis

**Affiliations:** 1 Ribeirão Preto College of Nursing, University of São Paulo, Ribeirão Preto 14040-902, Brazil; apedroso@usp.br (A.O.P.); mayramenegueti@usp.br (M.G.M.); egir@eerp.usp.br (E.G.); rkreis@eerp.usp.br (R.K.R.); 2 Faculty of Nursing, Federal University of Pará, Belém 66075-110, Brazil; elipinbt@gmail.com; 3 Department of Clinical Nursing, Federal University of Paraíba, João Pessoa 58051-900, Brazil; anacris.os@gmail.com

**Keywords:** HIV, acquired immunodeficiency syndrome, late diagnosis, nursing

## Abstract

Introduction: This study aimed to identify factors associated with late diagnosis and clinically monitor newly diagnosed HIV/AIDS patients. Method: Retrospective study, based on secondary data from a specialized unit at the Hospital das Clínicas da Faculdade de Medicina de Ribeirão Preto of the University of Sao Paulo. Data collection included sociodemographic, behavioral, clinical, and laboratory data of newly diagnosed HIV patients between 2015 and 2019. Data analysis was undertaken using inferential statistical tests. Results: A total of 314 individuals were newly diagnosed with HIV/AIDS, 86.6% (272) had a late diagnosis and 53.8% (169) were diagnosed very late. Using the adjusted odds ratio, we observed that bisexual and MSM patients were less likely to have a late diagnosis compared to straight patients. Individuals who entered through the emergency department and Outpatient Clinic had a lower chance of having a very late diagnosis compared to those diagnosed in the ward/inpatient unit. Having a higher education and university education were protective factors against having a very late diagnosis of HIV infection compared to elementary school education only. In addition, male patients were more likely to have a very late diagnosis compared to female patients. Conclusions: This study evidenced a high prevalence of late and very late diagnoses. Therefore, attention should be directed towards factors related to late and very late presentation.

## 1. Introduction

After four decades, human immunodeficiency virus (HIV) infection remains a global public health problem and continues to cause a high number of new infections. The Joint United Nations Programme on HIV/AIDS (UNAIDS) estimates that, in 2022, 1.3 million (1.0–1.7 million) people were newly infected with HIV [1]. The UNAIDS has formulated new 95-95-95 targets for treatment: 95% of people living with HIV know their serological status, 95% of those aware of their status are undergoing treatment, and 95% of individuals under treatment have suppressed viral loads. These targets are ambitious in their aim to eliminate the AIDS epidemic by 2030 and avert nearly 28 million new HIV infections and 21 million AIDS-related deaths [1].

To enhance early HIV diagnosis, novel HIV testing policies have been recommended by the World Health Organization [1] to optimize HIV testing services through expanded testing options and simplified service provision. These policies also seek to increase testing coverage and reinforce the adoption of HIV prevention and treatment services. Nevertheless, a substantial number of People Living with HIV (PLHIV) remain unaware of their infection or receive late diagnoses in countries with varying levels of development and income [2,3], thereby jeopardizing the achievement of epidemic-ending targets [2].

Late HIV diagnosis is characterized by an individual being diagnosed with HIV for the first time with a CD4 count < 350 cells/μL or due to an AIDS-defining event, irrespective of the CD4 cell count [4]. A proportion of these patients will have advanced HIV disease at the time of diagnosis (defined as CD4 count < 200 cells/μL or clinical AIDS).

The delay in diagnosing HIV infections increases the risk of transmission and the likelihood of treatment ineffectiveness or subsequent resistance. Simultaneously, it also raises the chances of treatment complications, opportunistic infections, and other non-infectious health issues. This results in higher rates of mortality and morbidity [5].

The first critical entry point to accessing care is receiving timely HIV testing and early HIV diagnosis [6]. The World Health Organization (WHO) has defined the early diagnosis of HIV infection as one of the priorities for controlling new infections [7].

Once early diagnosis is established, antiretroviral therapy (ART) can be started to maintain a CD4+ cell count above 500 cells/mm^3^ with undetectable viral load; in this way, a life expectancy similar to that of individuals not living with the virus can be observed [5].

In Canada, a study reported that in order to achieve the priority goal of the early diagnosis of HIV-infected individuals, it is essential not to miss opportunities in health services and to increase the availability of rapid tests to reach asymptomatic individuals with CD4+ cell counts above 500 cells/mm^3^ and without opportunistic diseases [5,8].

Chone et al. (2022), in Mozambique, observed that despite all the progress in combating the HIV epidemic in the region, some challenges remain, such as the high prevalence of late HIV diagnoses, even after the widespread availability and promotion of free diagnostic tests [9].

Late presentation continues to be a major challenge worldwide, despite all efforts made to prevent diseases and mortality associated with the late initiation of antiretroviral therapy (ART) and ensure continuous HIV treatment adherence [10]. In this context, a study in China showed a 57.6% increase (95% CI: 54.5–60.7%) in late HIV diagnosis. Over four years, there was an increase in the late presentation of HIV infection, with rates of 52.8% in 2017 and 61.2% in 2020 [11]. Research carried out in northeastern Brazil regarding the time of diagnosis showed that 59.1% of patients were diagnosed late (18.2% with a CD4+ T lymphocyte count of from 200 to 349 cells/mm^3^) or very late (40.9% with a CD4+ T lymphocyte count of less than 200 cells/mm^3^) [5].

Brazil has been striving to enhance its programmatic efforts, aiming to accelerate the Brazilian response to HIV/AIDS in alignment with the national targets that correspond to the Joint United Nations Programme on HIV/AIDS (UNAIDS) 95-95-95 goals and the global consensus for the elimination of the AIDS epidemic by 2030 [12]. However, a high number of people living with HIV/AIDS (PLWHA) present themselves to the public health system (SUS) for the first time with a CD4 count below 200 cells/mm^3^ [12]. Between 2012 and 2015, there was a decline in the trend of late presentation and advanced disease at healthcare facilities. However, from 2015 to 2019, the rate of late diagnoses remained stable, with a 29% increase from September 2022 [12]. Therefore, this study aimed to identify factors associated with late diagnosis and clinically monitor newly diagnosed HIV/AIDS patients.

## 2. Method

This is an observational, analytical study with a quantitative approach, retrospectively based on secondary data collected from the Electronic Patient Record (EPR).

The study was conducted at a specialized unit of a large tertiary hospital in the interior of São Paulo, the Hospital das Clínicas da Faculdade de Medicina de Ribeirão Preto da Universidade de São Paulo (HCFMRP-USP). The Specialized Unit for Infectious Diseases Therapy (UETDI) was inaugurated in 1996 with the aim of serving patients diagnosed with HIV/AIDS. It is considered a tertiary reference for the care of PLHIV in the Regional Health Department XIII (DRS XIII), which encompasses 26 municipalities. The healthcare is exclusively provided through the Unified Health System (SUS). The unit comprises three sectors: a ward, day hospital, and outpatient clinic for patients requiring medium- to high-complexity care. Brazil is divided into five major regions: the north, northeast, south, southeast and midwest. The southeast region, in which the state of São Paulo and the city of Ribeirão Preto are located, is the second smallest region in terms of area, has the largest number of inhabitants and the highest percentage of people living in cities, and is responsible for more than half of the Brazilian Gross Domestic Product (GDP) [13].

The study population consists of newly diagnosed cases of HIV/AIDS who sought initial care through outpatient appointments or hospitalization in the ward, whether referred from other services or not. The following inclusion criteria were adopted: aged 18 or older, of both sexes; available electronic medical records; diagnosed with HIV/AIDS in the past 6 months; this was the first visit to UETDI, either through outpatient care or ward admission, between 2015 and 2019. Pregnant women were excluded, as they are referred to the gynecology and obstetrics department for prenatal care, and it is not possible to collect data.

Data collection for sociodemographic, behavioral, clinical, and laboratory variables was carried out using patient record entries. The chosen period was from 2015 to 2019, as electronic medical records were implemented in 2015, replacing paper documents. A review of all patient records during the selected period who initially received care through the unit’s outpatient clinic, and later through the ward, was conducted.

The following variables were selected: (a) sociodemographic: sex at birth (female/male), sexual orientation, date of birth, age, nationality (place of birth) and origin (municipality of residence of the individual), marital status, and education level; (b) behavioral: smoking, alcohol consumption, illicit drug use, types of illicit drugs, and duration of illicit drug use; (c) clinical–epidemiological: date of first visit, duration of HIV infection diagnosis, use of antiretroviral therapy (ART), duration of ART use, associated comorbidities, (d) death criteria (death report mentioning AIDS or HIV and cause of death associated with immunodeficiency—without classification by another criteria after investigation; date of death; reason and location), described according to the B20–B24 codes of the International Classification of Diseases (ICD-10) and recorded in the electronic medical records.

The definition of late HIV diagnosis encompasses a CD4 count of <350 cells/mm^3^ or an AIDS-defining event. Additionally, a very late diagnosis corresponds to a CD4 count of <200 cells/mm^3^, representing a subset of the late diagnosis group [4]. For analytical purposes, the data were stratified into two distinct groups: the Late Diagnosis group (CD4 < 350 cells/mm^3^) and the Very Late Diagnosis group (CD4 < 200 cells/mm^3^).

The data were entered into an Excel spreadsheet for Windows, with double entry and data validation to identify possible typing errors. Subsequently, the spreadsheet was imported into the Statistical Package for the Social Sciences (SPSS), version 25.0 for Windows, for data analysis. Descriptive statistics, such as mean, median, standard deviation, minimum, and maximum, were used for continuous variables, while relative and absolute frequencies were used for categorical variables. To calculate the crude odds ratio, we selected the variables that could be considered factors associated with late and very late diagnosis. Variables that were statistically significant (*p* value < 0.05) in calculating the crude odds ratio in at least one category were included in the final model using the stepwise method to estimate the adjusted odds ratio.

The present research project was submitted and approved by the Research Ethics Committee of the Ribeirão Preto College of Nursing, University of São Paulo, under protocol number 4,143,945.

## 3. Results

A total of 1120 HIV/AIDS patients were treated at the tertiary hospital during the study period, of whom 314 (28%) were newly diagnosed with HIV infection. Among the total, 86.6% (272) had a late diagnosis, 208 had a CD4+ T-cell count below 350 cells/mm^3^ and 64 had a CD4+ T-cell count greater than 350, but had an opportunistic disease (late diagnosis), and 53.8% (169) had a very late diagnosis.

**Figure 1:**
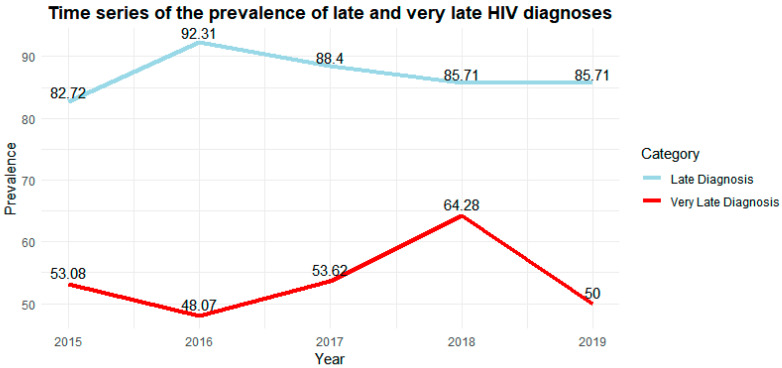
Time series of the prevalence of late and very late diagnosis of HIV infection. Ribeirão Preto, SP, Brazil, 2023. Source: Study Data, Ribeirão Preto/SP, 2023.

Figure 1 displays the prevalence of late and very late diagnoses within the period from 2015 to 2019.

Table 1 presents the sociodemographic and behavioral variables of patients with late and very late diagnosis of HIV infection.

Regarding the clinical and outcome variables, Table 2 presents the main results.

To calculate the crude odds ratio, we selected the variables that could be considered factors associated with late and very late diagnosis. Using the adjusted odds ratio, we observed that bisexual patients and men who have sex with men (MSM) were less likely to have a late diagnosis compared to straight patients, as shown in Table 3.

Furthermore, individuals who entered through the emergency department and outpatient clinic had a lower chance of having a very late diagnosis compared to those diagnosed in the ward/inpatient unit. Having a higher education and university education were protective factors against a very late diagnosis of HIV infection compared to elementary school education. In addition, male patients were more likely to have a very late diagnosis compared to female patients. All data are presented in Table 4.

## 4. Discussion

This study revealed that although patients seen in the initial care setting arrive as newly diagnosed cases of HIV, the majority can be considered as having a late diagnosis. Of all patients, 86.6% (272) had a late diagnosis, 208 had a CD4+ T-cell count below 350 cells/mm^3^, 64 had a CD4+ T-cell count greater than 350 but had an opportunistic disease (late diagnosis), and 53.8% had a CD4+ T-cell count below 200 cells/mm^3^ (very late diagnosis). This high rate is similar to a study conducted in a state in Northeast Brazil, which reported 59.1% in 2017 [5], and to other countries, such as China, where a percentage greater than 60% was observed from 2015 to 2016 [14], and Ethiopia, with 68.8% in 2014 [15]. These data are alarming when compared to national figures, which identified that 42% [16] and 48% [5] of diagnosed individuals had a CD4+ T-cell count below 350 cells/mm^3^ in 2015 and 2022, respectively.

Certain sociodemographic factors, such as educational level, may be directly linked to access to healthcare, impacting the prevalence of late or very late diagnosis [17]. In this study, it was found that 56.3% of patients detected with a CD4+ T-cell count below 200 cells/mm^3^ had only a primary education, similar to findings from a European cohort study conducted from 1996 to 2013, which identified that lower educational attainment was associated with a higher chance of individuals having lower CD4+ T-cell counts and a lower chance of viral suppression [18]. Another study, conducted in Minas Gerais, Brazil, between 2012 and 2018, also found evidence of an association between lower education levels and a lower chance of achieving viral suppression [19].

Regarding sex at birth, this study found that being male was a factor associated with very late diagnosis, which is consistent with previous research. In Nigeria, a high percentage of men were also identified as receiving a late HIV diagnosis (90.1% vs. 83.3% for women; *p* < 0.001) or having advanced disease (70.4% vs. 59.2% for women; *p* < 0.001) [20]. Another study conducted in Salvador, Brazil, found that being male increased the chances of delayed diagnosis, with an adjusted odds ratio of 3.02; 95% CI, 2.0–4.6 [21]. According to data from the Brazilian Ministry of Health, when stratified by sex (at birth), men stand out compared to women. Regarding late diagnosis (CD4+ T-cell count < 350 cells/mm^3^), as of September 2022, the proportion between sexes was 49% male and 46% female. For very late diagnosis (CD4+ T-cell count < 200 cells/mm^3^), this proportion was 29% of men and 27% of women [12].

The higher prevalence observed in males may be related to advances and achievements in HIV response policies for women [22] and pregnant women, such as individual or group counseling, family planning, regular gynecological care, prenatal care, HIV testing centers, and maternity wards [23]. Therefore, access to screening for infections like HIV serves as a facilitator for preventive measures and timely diagnosis. A study conducted in Ghana in 2018 confirmed that the prevalence of HIV among pregnant women was lower compared to the general population [24]. On the other hand, the testing policy during prenatal care has certain effects on the male population, as women discover the presence of HIV infection during pregnancy or through illness, leading to this diagnosis being discovered by their male partners [25].

A study conducted in the southern region of Brazil in 2019 showed that low risk perception is extremely relevant. Over 90% of interviewed individuals identified themselves as having low or no risk of HIV infection, and over 30% had never been tested for HIV because they did not consider themselves at risk or saw no reason to get tested [26].

Regarding the mode of entry, entering the healthcare facility through the ward was associated with late diagnosis compared to patients who came for outpatient consultations. This finding is similar to a study conducted in Malawi in 2020, which found that 53.3% of hospitalized patients newly diagnosed with HIV had a CD4+ T-cell count below 200 cells/mm^3^, and 32% already had counts below 100 cells/mm^3^ [27]. Another study conducted in South Africa between 2014 and 2015 showed that patients entering the healthcare system through inpatient settings were more likely to have late HIV diagnosis compared to those entering through outpatient settings. Hospitalized patients had very low CD4+ T-cell counts, with a median of 37 cells/mm^3^, which was higher than the counts observed in outpatient patients [28].

It is known that ART should be started immediately for all PLHIV, as soon as they are diagnosed, regardless of their clinical or immunological status. This has been a recommendation of the Ministry of Health since December, 2013 [29]. However, in this study, there was a gap between the diagnosis of HIV and the start of ART. This may be due to the fact that many patients were diagnosed late and had opportunistic diseases, leading to a delay in starting ART, since in many cases it is necessary to treat associated diseases in order to subsequently treat the HIV infection, which is not part of the protocol. This can lead to non-adherence to treatment. This shows the importance of interventions by the health team through systematic and personalized follow-up, particularly for this patient profile.

Even though ART initiation is considered to be a consequence of late diagnosis, the literature highlights the positive impact of the immediate initiation of ART after HIV diagnosis [30]. Viral suppression through ART has both individual and collective health effects, as the treatment serves to prevent new infections [31]. In the United Kingdom, a significant number of people living with HIV achieved viral suppression reaching 97%, further emphasizing the effectiveness of ART treatment [32].

A retrospective cohort study conducted in China between 2006 and 2020 demonstrated that the early initiation of ART in people living with HIV significantly reduced the mortality rate in this population, directly impacting overall mortality rates. However, the same study found that factors such as being male, having a very late HIV diagnosis, initiating ART after 12 months of diagnosis, and virologic failure (detectable viral load after 6 months of treatment initiation or modification) led to a higher mortality rate [29,33].

Despite advances in treatment and care for people living with HIV, mortality rates remain high in cases of late diagnosis [34]. A study conducted in Colombia between 2009 and 2014 with people living with HIV found that 80% of admissions to intensive care units were associated with opportunistic infections, with 57% attributed to respiratory failure as the leading cause of death in this population [35]. Similarly, another study conducted in French hospitals between 1997 and 2020 showed that although the proportion of admissions for HIV-diagnosed patients and the rate of opportunistic infections significantly decreased, the reasons for admission remained consistent over time, with respiratory failure being the main cause [36].

A similar study in Korea between 2004 and 2018 demonstrated that the development of opportunistic diseases within 6 months after HIV diagnosis was more common in men. Additionally, individuals had overall higher mortality rates when they had AIDS-defining illnesses and AIDS-associated cancers [37]. It is important to highlight the significance of treatment adherence, as higher mortality rates are observed in people living with HIV who are not adequately treated [38].

Late HIV diagnosis has consequences for both individual and public health [39], as well as economic implications [15]. Therefore, strategies are needed to improve timely diagnosis, such as targeted campaigns for prevention and diagnosis, the promotion of early testing, and care [40]. Overcoming barriers such as difficulties in accessing tests, a lack of awareness of HIV-related risks and diseases, stigma, and prejudice can significantly reduce cases of late diagnosis [41].

The implementation of effective strategies that enable the early detection of HIV diagnosis is clearly necessary, as these could lead to a reduction in disease transmissibility and consequently decrease the likelihood of late diagnoses [42]. In Spain, in 2019, it was found that 16% of patients diagnosed with HIV had missed opportunities for diagnosis in the previous 5 years, as they sought medical attention for other associated illnesses but did not undergo testing for HIV [42]. Similarly, a study in Canada from 2001 to 2014 showed that from 7 to 14% of individuals had one or more missed opportunities for HIV diagnosis, even in a setting with unrestricted healthcare [8].

AIDS-related mortality is strongly associated with late presentation, particularly in the first year following HIV diagnosis. It is clear that one of the causes is the missed opportunities for serological testing when individuals seek healthcare services prior to diagnosis [43].

As a retrospective study, this study is subject to limitations that include the need to obtain secondary data from electronic medical records, which means that some information could not be further explored, such as the monitoring and clinical outcomes of patients who were transferred to other HIV and AIDS reference centers within and outside Ribeirão Preto, as well as inadequate completion of the available electronic data collection instruments during the initial assessment, resulting in a lack of important social and behavioral information. Furthermore, it was not possible to be sure that there were no cases of acute infection among patients with late and very late diagnosis. However, to minimize this limitation, we used the medical records to verify that there was no record that the patients had recently been exposed to risk. Another limitation was the emergence of the COVID-19 pandemic, which may have influenced some current significant results in the care and treatment of patients diagnosed with HIV, as the data collection period extended until 2019.

## 5. Conclusions

This study evidenced a high prevalence of late and very late diagnoses. Therefore, attention should be directed towards factors related to late and very late presentation, such as being a male adult with a lower education level, as evidenced in this study. It is known that very late presentation is directly correlated with higher mortality rates, as also highlighted by this study. Clinical outcomes are of the utmost importance when evaluating diagnosis and treatment adherence, as they reflect the barriers to prevention and the quality of care provided to this population.

The implementation of new protective measures and campaigns is essential in reducing cases of very late presentation, not only among high-risk populations but also among those who perceive themselves as having low or no risk of infection. However, further research needs to be conducted to fill the gaps identified in this study, including the impact of the COVID-19 pandemic on the diagnosis and treatment of these individuals and professional training, which can be considered a barrier to timely diagnosis and treatment compliance.

## Figures and Tables

**Table 1:** Sociodemographic and behavioral variables of patients with late and very late diagnosis of HIV infection. Ribeirão Preto, SP, Brazil, 2022.

Variables		Late Diagnosis (CD4 < 350 and/or with an AIDS-Defining Event)			Very Late Diagnosis (CD4 < 200)		
		No	Yes	* *p*	No	Yes	* *p*
		*n* = 24 (%)	*n* = 272 (%)		*n* = 127 (%)	*n* = 169 (%)	
Age							
	Mean (Standard Deviation)	35.6 (12.9)	38.8 (12.6)	0.24 **	36.9 (12.8)	39.7 (12.3)	0.055 **
Sex (at birth)							
	Female	11 (45.8)	67 (24.6)	0.03	41 (32.3)	37 (21.9)	0.047
	Male	13 (54.2)	205 (75.4)		86 (67.7)	132 (78.1)	
Nationality							
	Ribeirão Preto	4 (16.7)	54 (19.9)	0.85	26 (20.5)	32 (18.9)	0.17
	Ribeirão Preto Region	5 (20.8)	42 (15.4)		24 (18.8)	23 (13.6)	
	State of São Paulo	8 (33.3)	104 (38.2)		51 (40.2)	61 (36.2)	
	Outside the state of São Paulo	7 (29.2)	72 (26.5)		26 (20.5)	53 (31.3)	
Origin							
	Ribeirão Preto	6 (25.0)	99 (37.2)	0.60	41 (32.8)	64 (38.8)	0.11
	Ribeirão Preto Region	06 (25.0)	61 (22.9)		29 (23.2)	38 (23.0)	
	State of São Paulo	12 (50.0)	101 (38.0)		55 (44.0)	58 (35.2)	
	Outside the State of São Paulo	0 (0)	5 (1.9)		0 (0)	5 (3.0)	
Marital State							
	Single	16 (66.7)	146 (53.9)	0.06	72 (56.7)	90 (53.5)	0.72
	Married/Common-law/living as married	4 (16.7)	85 (31.4)		38 (29.9)	51 (30.4)	
	Widower	3 (12.4)	9 (3.3)		6 (4.7)	6 (3.6)	
	Separated/Divorced	1 (4.2)	31 (11.4)		11 (8.7)	21 (12.5)	
Education Level							
	Elementary School	12 (52.2)	135 (50.2)	0.213	53 (42.4)	94 (56.3)	0.036
	High School	6 (26.1)	99 (36.8)		48 (38.4)	57 (34.1)	
	University	3 (13.0)	29 (10.8)		20 (16.0)	12 (7.2)	
	Illiterate	2 (8.7)	6 (2.2)		4 (3.2)	4 (2.4)	
Sexual Orientation							
	Straight	11 (50.0)	168 (67.2)	0.04	73 (61.3)	106 (69.4)	0.34
	Bisexual	5 (22.7)	15 (6.0)		10 (8.4)	10 (6.5)	
	Homosexual	2 (9.1)	41 (16.4)		24 (20.2)	19 (12.4)	
	MSM	4 (18.2)	25 (10.0)		12 (10.1)	17 (11.1)	
	Others	0 (0)	1 (0.4)		0 (0)	1 (0.6)	
Smoker							
	Yes	17 (70.8)	166 (61.5)	0.51	77 (61.1)	106 (63.1)	0.81
	No	7 (29.2)	104 (38.5)		49 (38.9)	62 (36.9)	
Drug Use							
	Yes	12 (50.0)	97 (36.1)	0.191	46 (36.2)	63 (37.9)	0.80
	No	12 (50.0)	172 (63.9)		81 (63.8)	103 (62.1)	

Source: Study Data, Ribeirão Preto/SP, 2022. * Fisher’s exact test ** Student’s *t*-test.

**Table 2:** Clinical and outcome variables of patients with late and very late diagnosis among people diagnosed with HIV/AIDS infection. Ribeirão Preto, SP, Brazil, 2022.

Variable		Late Diagnosis (CD4 < 350 and/or AIDS-Defining Event)			Very Late Diagnosis (CD4 < 200)		
		No	Yes	* *p*	No	Yes	* *p*
		*n* = 24 (%)	*n* = 272 (%)		*n* = 127 (%)	*n* = 169 (%)	
Admission							
	Hospital Ward—Inpatient	6 (25.0)	122 (44.9)	0.111	22 (17.3)	106 (62.7)	<0.001
	Emergency Care	15 (62.5)	112 (41.2)		86 (67.7)	41 (24.3)	
	Outpatient Clinic	3 (12.5)	38 (13.9)		19 (15.0)	22 (13.0)	
Extrapulmonary tuberculosis							
	Yes	0 (0.0)	12 (4.4)	0.608	02 (1.6)	10 (5.9)	0.076
	No	24 (100.0)	260 (95.6)		125 (98.4)	159 (94.1)	
Histoplasmosis							
	Yes	0 (0.0)	08 (2.9)	1.000	0 (0.0)	08 (4.7)	0.012
	No	24 (100.0)	264 (97.1)		127 (100.0)	161 (95.3)	
Neurotoxoplasmosis							
	Yes	0 (0.0)	25 (9.2)	0.241	03 (2.4)	22 (13.0)	0.001
	No	24 (100.0)	247 (90.8)		124 (97.6)	147 (87.0)	
Pneumocystosis							
	Yes	0 (0.0)	02 (0.7)	1.000	0 (0)	02 (1.2)	0.508
	No	24 (100.0)	270 (99.3)		127 (100.0)	167 (98.8)	
Cytomegalovirus							
	Yes	0 (0)	32 (11.8)	0.089	01 (0.8)	31 (18.3)	<0.001
	No	24 (100.0)	240 (88.2)		126 (99.2)	138 (81.7)	
Disseminated cryptococcosis							
	Yes	0 (0)	11 (4.0)	0.609	01 (0.8)	10 (5.9)	0.027
	No	24 (100.0)	261 (96.0)		126 (99.2)	159 (94.1)	
Kaposi’s sarcoma							
	Yes	0 (0)	93 (34.2)	<0.001	73 (57.5)	20 (11.8)	<0.001
	No	24 (100.0)	179 (65.8)		54 (42.5)	149 (88.2)	
Herpes							
	Yes	0 (0.0)	07 (2.6)	1.000	05 (3.9)	02 (1.2)	0.143
	No	24 (100.0)	265 (97.4)		122 (96.1)	167 (98.8)	
Lymphoma							
	Yes	0 (0.0)	07 (2.6)	1.000	02 (1.6)	05 (3.0)	0.703
	No	24 (100.0)	265 (97.4)		125 (98.4)	164 (97.0)	
ART							
	Yes	02 (8.3)	52 (19.1)	0.27	15 (11.8)	39 (23.1)	0.01
	No	22 (91.7)	220 (80.9)		112 (88.2)	130 (76.9)	
Death							
	Yes	1 (4.2)	36 (13.2)	0.07	09 (7.1)	28 (16.6)	0.003
	No	15 (62.5)	164 (60.3)		89 (70.1)	90 (53.2)	
	Transfer	03 (12.5)	54 (19.9)		17 (13.4)	40 (23.7)	
	Loss of Segment	05 (20.8)	18 (6.6)		12 (9.4)	11 (6.5)	

Source: Study Data, Ribeirão Preto/SP, 2022. * Fisher’s exact test.

**Table 3:** Factors associated with late diagnosis of HIV infection according to the logistic regression model. Ribeirão Preto, SP, Brazil, 2022.

CD4 < 350 and/or with an AIDS-Defining Event	Crude Odds Ratio	CI95% *	*p* Value **	Adjusted Odds Ratio	CI95% *	*p* Value ***
Age (per 1 year older)	1.02	0.986–1.059	0.242			
Sex (at birth)						
Female	Comparison Category			Comparison Category		
Male	2.59	1.11–6.05	0.028	3.71	0.968–14.201	0.056
Admission						
Hospital Ward—Inpatient	Comparison Category			Comparison Category		
Emergency Care	0.42	0.177–0.993	0.048	0.42	0.138–1.253	0.119
Outpatient Clinic	1.14	0.323–3.997	0.842	0.61	0.129–2.876	0.531
Smoker						
Yes	Comparison Category					
No	1.52	0.610–3.794	0.368			
Drug Use						
Yes	Comparison Category					
No	1.77	0.767–4.099	0.180			
Sexual Orientation						
Straight	Comparison Category					
Bisexual	0.22	0.070–0.669	0.008	0.11	0.022–0.521	0.006
Homosexual	1.96	0.441–8.718	0.376	0.72	0.115–4.512	0.726
MSM ^&^	0.5	0.157–1.594	0.241	0.5	0.349–0.949	0.043
Others	1.0			1.0		
Education Level						
Elementary School	Comparison Category					
High School	1.65	0.629–4.322	0.308			
University	0.81	0.226–2.878	0.739			
Illiterate	0.24	0.046–1.261	0.092			
Marital State						
Single	Comparison Category					
Married/Common-law/Living as married	2.28	0.758–6.890	0.142	1.24	0.337–4.599	0.743
Widower	0.24	0.060–0.956	0.043	0.36	0.067–1.959	0.238
Separated/Divorced	2.97	0.387–22.773	0.295	2.47	0.275–36.840	0.419

Source: Study Data, Ribeirão Preto/SP, 2022. * CI = 95% Confidence Interval; ** logistic model bivariate; *** logistic model multivariate; ^&^ men who have sex with men (MSM).

**Table 4:** Factors associated with very late diagnosis of HIV infection according to the logistic regression model. Ribeirão Preto, SP, Brazil, 2022.

CD4 < 200	Crude Odds Ratio	CI95% *	*p* Value **	Adjusted Odds Ratio	CI95% *	*p* Value ***
Age (per 1 year older)	1.02	0.999–1.037	0.056			
Sex (at birth)						
Female	Comparison Category			Comparison Category		
Male	1.70	1.010–2.863	0.046	2.01	1.049–3.854	0.035
Admission						
Hospital Ward—Inpatient	Comparison Category			Comparison Category		
Emergency Care	0.15	0.092–0.255	<0.001	0.09	0.047–0.166	<0.001
Outpatient Clinic	0.85	0.439–1.649	0.632	0.22	0.097–0.481	<0.001
Smoker						
Yes	Comparison Category					
No	0.92	0.571–1.479	0.728			
Drug Use						
Yes	Comparison Category					
No	0.93	0.575–1.499	0.761			
Sexual Orientation						
Straight	Comparison Category					
Bisexual	0.76	0.306–1.896	0.559			
Homosexual	0.56	0.291–1.082	0.085			
MSM ^&^	1.11	0.510–2.434	0.785			
Others	1.0					
Education Level						
Elementary School	Comparison Category			Comparison Category		
High School	0.83	0.513–1.346	0.452	0.54	0.291–0.989	0.046
University	0.41	0.190–0.867	0.020	0.27	0.108–0.709	0.007
Illiterate	0.74	0.182–3.027	0.678	1.83	0.403–8.309	0.433
Marital State						
Single	Comparison Category					
Married/common-law/living as married	1.02	0.618–1.687	0.936			
Widower	0.75	0.235–2.373	0.621			
Separated/Divorced	1.51	0.698–3.251	0.296			

Source: Study Data, Ribeirão Preto/SP, 2022. * CI = 95% Confidence Interval; ** logistic model bivariate; *** logistic model multivariate; ^&^ men who have sex with men (MSM).

## Data Availability

Not applicable.
